# Insights to the neural response to food cues in class III compared with class I and II obese adults using a sample of endometrial cancer survivors seeking weight loss

**DOI:** 10.1038/s41387-020-0124-7

**Published:** 2020-06-15

**Authors:** Nora L. Nock, Huangqi Jiang, Lauren Borato, Jay Alberts, Anastasia Dimitropoulos

**Affiliations:** 1 Departments of Population and Quantitative Health Sciences, Case Western Reserve University, Cleveland, OH USA; 2 Case Comprehensive Cancer Center, Cleveland, OH USA; 3 Psychological Sciences, Case Western Reserve University, Cleveland, OH USA; 4 Department of Biomedical Engineering, Cleveland Clinic, Cleveland, OH USA

**Keywords:** Cancer, Obesity

## Abstract

**Background:**

The rates of severe or Class III obesity (BMI ≥ 40.0 kg/m^2^) and endometrial cancer (EC) incidence and mortality have been increasing significantly in the United States. Adults with severe obesity are more likely to die and women with severe obesity have a higher risk of EC development and mortality than those with Class I/II obesity (BMI: 30–<40 kg/m^2^). However, no prior studies have evaluated the neural response to food cues by obesity severity/class in adults with or without cancer.

**Methods:**

We conducted a functional magnetic resonance imaging visual food cue task in 85 obese Stage I EC survivors who were seeking weight loss in a lifestyle intervention at baseline. We evaluated the neural response to high-calorie vs. non-food images after an overnight fast (fasted state) and after eating a standardized meal (fed state), and grouped patients by obesity class (Class I/II: *n* = 38; Class III: *n* = 47).

**Results:**

In the fasted state, we found increased activation in several regions including the dorsolateral prefrontal cortex (DLPFC) in Class III and Class I/II patients (whole brain cluster corrected (WBCC), *p* < 0.05), which was significantly higher in Class III vs. Class I/II (*p* < 0.05). We found decreased activation in the insula in the fasted state, which was significantly lower in Class I/II vs. Class III (*p* = 0.03). In the fed state, we found increased activation in the DLPFC in Class III and Class I/II (WBCC, *p* < 0.05). The increased activation in cognitive control/inhibition regions (DLPFC) is consistent with the summative literature; however, the decreased activation in taste information processing regions (insula) was unexpected.

**Conclusions:**

Our results provide novel insights on food cue response between different classes of obesity and highlight the importance of targeting the DLPFC in weight loss interventions, particularly in severely obese patients. Additional studies examining food-related neural circuitry between different classes of obesity are needed.

## Introduction

Severe or Class III obesity (body mass index (BMI) ≥ 40 kg/m^2^)^1,2^ is increasing in several developed nations including the United States^3,4^, where adults with a BMI exceeding 40 kg/m^2^ or 50 kg/m^2^ has increased more than fourfold and tenfold, respectively, in the past several years^3^. A pooled analysis of 20 prospective studies found that Class III obese compared with normal weight (BMI: 18.5 to <25 kg/m^2^) adults had significantly higher mortality rates of 2.57 (95% confidence interval (CI): 2.41–2.74) overall and 1.40 (95% CI: 1.31–1.51) for every 5 unit increase in BMI^5^. Furthermore, adults with a BMI of 40–44.9, 45–49.9, 50–54.9, and 55–59.9 kg/m^2^ have an estimated 6.5 (95% CI: 5.7–7.3), 8.9 (95% CI: 7.4–10.4), 9.8 (95% CI: 7.4–12.2), and 13.7 (95% CI: 10.5–16.9) life years lost^5^.

Cancer is the second leading cause of death in the United States^6^ and there are currently more than 15.5 million cancer survivors living in the United States, with a projected estimate of 20 million by 2026^7^.

Uterine cancer is the fourth leading cause of cancer in women in the United States, with ~61,880 new cases estimated for 2019 and, the sixth leading cause of cancer death with 12,160 deaths estimated for 2019^8^. Currently, there are ~757,190 uterine cancer survivors and by 2026 it is projected that 942,670 uterine cancer survivors will be living in the United States^7^. Over 95% of all uterine cancers occur in the endometrium^9^; thus, most of these women are endometrial cancer survivors.

Although the incidence and mortality rates of most cancers appear to be stable or decreasing, the incidence and mortality rates of uterine cancer have been rising at a rate of ~2% per year^6^. In particular, the incidence rate of Type I (estrogen dependent) endometrial cancer is increasing^10–12^ and this cancer type appears to be driving the overall rise in uterine cancer incidence rates observed.

Higher BMI has been associated with an increased risk of development and mortality of several cancers including endometrial cancer^13–16^. Women with obesity (BMI ≥ 30.0 kg/m^2^) have a 1.7- to 4.5-fold greater risk of developing endometrial cancer compared with those who are of normal weight^17–21^. Moreover, endometrial cancer patients have the highest risk of death among all of the obesity-associated cancers, with a 2.5-fold increased risk of death for women with Class I (BMI: ≥30.0–<35.0 kg/m^2^) up to a 6-fold increased risk of death in women with Class III obesity^10^.

Obesity and cancer are both complex diseases with numerous risk factors; however, studies are beginning to unravel the underlying mechanisms including several hormonal, metabolic and inflammatory factors that link the two diseases^22,23^. Obesity itself likely involves the interplay of behavioral, social, environmental, genetic, and neurological factors^24^. In particular, obesity involves the disruption to neurological reward systems, which can override homeostatic energy systems leading to overeating of high-calorie foods that have intensely rewarding properties^25,26^. In adults with obesity, high-calorie food stimuli may possess greater potency for activating the reward system and may trigger excessive motivation to non-homeostatic eating^26^. In particular, women with obesity and without cancer shown pictures of high-calorie foods have been found to have increased activation in the dorsal striatum^27,28^, a brain region implicated in reward anticipation and cue-induced drug-craving and drug-seeking^29^. In addition, higher activation in women with obesity without cancer compared with normal weight was observed in regions associated with taste information processing (e.g., anterior insula and lateral orbitofrontal cortex (OFC)), motivation (e.g., OFC), and memory (e.g., posterior cingulate)^29–32^. Other areas involving attention (e.g., anterior cingulate cortex), motor processes (e.g., precentral gyrus and cerebellum), and general reward processing (e.g., striatum, amygdala, and OFC) have also been reported to have increased activation in non-cancer adults with obesity compared with those who are of normal weight^27,30,33,34^.

Information on the neural response to visual food cues in adults with severe obesity, particularly in comparison with other levels or classes of obesity, is limited. Most of the studies involving severely obese adults have been conducted after bariatric surgery was performed and weight loss occurred. Nevertheless, these studies suggest that the brain activity patterns in severely obese adults may be different than obese patients after bariatric surgery and in comparison with normal weight women. For example, Frank et al.^35^ found that severely obese women (*n* = 11) had lower hypothalamic activation during presentation of high-calorie vs. low-calorie food pictures after consuming a 246 kcal drink compared with normal weight women (*n* = 11) and previously severely obese women ~3 years, on average, after Roux-en Y gastric bypass (RYGB) surgery (*n* = 9)^35^. Other studies have shown reduced activation in mesolimbic and dorsolateral frontal regions 1 month after RYGB surgery compared with preoperative responses, which were more pronounced in fasted compared with fed (postprandial) states^36–38^.

To our knowledge, we are the only group who has evaluated responses to visual food cues in cancer survivors with obesity. In a small pilot study of endometrial cancer patients enrolled in a larger behavioral lifestyle intervention, which aimed to improve diet quality by increasing the intake of low-calorie/nutrient-rich foods (e.g., fruits and vegetables) and decreasing the intake of high-calorie/nutrient-weak foods (e.g., chips and sweets)^39^, we found that endometrial cancer survivors with Class II obesity, on average (mean BMI of 35.8 kg/m^2^), generally had similar responses to visual food cues as the wider population of obese adults without cancer but we also observed some unanticipated findings^40^. In the pre-meal condition at baseline, we found significant increased activation when comparing high-calorie vs. non-food cues in several regions including the dorsolateral prefrontal cortex (DLPFC), OFC, and the medial frontal gyrus (MFG), and an unexpected decreased activation in the anterior cingulate when comparing high-calorie with low-calorie food cues^40^. In the post-meal condition at baseline, we found significant increased activation in the thalamus, posterior cingulate, and precuneus when comparing high-calorie with low-calorie food cues^40^. As our sample size was very small in this study, we were not able to stratify by obesity class.

To our knowledge, no prior studies have examined the neural response to visual food cues by obesity severity or class, which could help enhance behavioral interventions by targeting specific brain regions that differ between obesity classes. Therefore, we aimed to evaluate the response to visual food cues in a relatively large population of obese women spanning the range of obesity classes (Class I–III) using a sample of endometrial cancer survivors with obesity, who were seeking weight loss as part of a lifestyle intervention at baseline. We hypothesized that women with Class III compared with Class I and II obesity would have greater activation in regions associated with food reward and motivation in response to high-calorie compared with non-food images, which would persist after eating a meal.

## Methods

### Study population

The study population consisted of 85 obese (BMI ≥ 30.0 kg/m^2^) Stage I endometrial cancer patients enrolled in a lifestyle intervention at baseline (and before randomization) at University Hospitals Case Medical Center and the Cleveland Clinic (ClinicalTrials.gov Identifier: NCT01870947). The protocol was approved by the Institutional Review Board of University Hospitals Case Medical Center (UHCMC) and the Cleveland Clinic. All patients provided informed written consent.

### fMRI procedures

The protocol has been described previously^41^; however, we include the relevant details herein. Patients were instructed to fast overnight and underwent structural and functional magnetic resonance imaging (MRI) scans from ~11:00 a.m. to 1:00 p.m. Patients were provided a 1000 kcal standardized luncheon meal prepared by the Dahms Clinical Research Unit (DCRU) Metabolic Kitchen at University Hospitals. Patients were instructed to eat to satiation and any remaining food was returned to the DCRU to be weighed, to provide an estimate of the amount of food consumed. Approximately 25–30 min after the first scan, patients underwent the second set of functional scans (post-meal, fed state).

Prior to scanning, a food preference assessment was administered, whereby participants rated photograph flash cards of 74 foods (PCI Education, San Antonio, TX, USA) that included fruits, vegetables, desserts, meats, snacks, breads, and pastas on a five-point Likert scale from “dislike” (1) to “like” (5). Food preference (“liking”) ratings for high-calorie (e.g., cakes, cookies, potato chips, änd hot dogs) and low-calorie (e.g., fruits and vegetables) foods were compared between groups. Immediately before each scan, participants were asked to answer the following question: “How hungry do you feel?”, by marking a vertical line on a Visual Analog Scale anchored with ends labeled “I am not hungry at all” and “I have never been more hungry^42^.”

### fMRI experimental visual food cue task

Changes in blood oxygen level-dependent (BOLD) contrast were measured using a blocked design, perceptual discrimination task, whereby patients indicated whether side-by-side color images of high-calorie food (e.g., cake, doughnuts, chips, and fries), low-calorie food (fresh fruits and vegetables), and non-food objects (e.g., assorted furniture) were the “same” or “different” using a button press^43^. The side-by-side images were from the same category (i.e., high-calorie, low-calorie, or non-food) and each image was presented only once. Images were presented in two runs per session (pre-meal and post-meal) and comprised eight blocks (21 s each with a 14 s rest between blocks) with six image pairs per block. Each run presented blocks of high-calorie foods, low-calorie foods, and non-food in a counterbalanced order. Stimulus duration was set at 2250 ms with a 1250 ms inter-stimulus interval. The same/different tasks were selected to ensure participants were attending to the stimuli.

### fMRI data acquisition and analysis

Data were acquired on a Wide-Bore Verio or Skyra 3.0 T MRI scanner (Siemens Medical Solutions, Malvern, PA) with a bore width of 70 cm and 550 lb weight capacity equipped with a 12-channel receiver head coil, an audio/visual system (Avotec, Inc., Stuart, FL, USA), and an integrated four button response device (Lumina) at UHCMC and the Case Center for Imaging Research. Stimulus presentation was controlled by a computer synchronized to the 3.0 T operation using EPRIME (Psychology Software Tools, Inc.; www.pstnet.com/eprime). Functional images were acquired using a gradient- echo single-shot echo-planar sequence over 34 contiguous axial sequence slices aligned parallel to AC-PC plane with an in-plane resolution of 3.4 × 3.4 × 3 mm (Repetition Time (TR) = 1950, Time to Echo (TE) = 22 ms, flip angle = 90°). BOLD activation data were acquired during two runs (5:01 min, 157 Echo-planar imaging (EPI)) per session. Two-dimensional (2D) T1-weighted (T1) radio frequency spoiled gradient echo images (TR = 300, TE = 2.47 ms, Field of View (FOV) = 256, matrix = 256 × 256, flip angle = 60°, Number of Excitations (NEX) = 2) in the same locations as the echo-planar data for in-plane registration and high-resolution three-dimensional (3D) structural images (3D Magnetization-Prepared Rapid Acquisition Gradient Echo (MPRAGE), contiguous, sagittal acquisition, 176 images with 1 mm isotropic voxels, TR = 2500, TE = 3.52 ms, Inversion Time (TI) = 1100, FOV = 256, matrix = 256 × 256, flip angle = 12°, NEX = 1) for Talairach normalization and anatomical overlay were collected during the pre-meal session.

Image processing, statistical analyses, and tests of statistical significance were performed using BrainVoyager 20.2 (Brain Innovation, Maastricht, The Netherlands). Preprocessing steps included trilinear 3D motion correction, 2D spatial smoothing with a Gaussian filter with full width half-maximum of 7 mm and high-pass filter temporal smoothing/linear trend removal. High-resolution functional 2D images were aligned to 3D anatomical images for display and localization using piecewise linear transformation into a proportional 3D grid defined by Talairach and Tournoux^44^, and were co-registered with the high-resolution 3D data set and resampled to 3 mm^3^ voxels. Motion correction parameters were added to the design matrix and any movement >2 mm along any *x*-, *y*-, or *z*-axis was discarded (<1% was discarded).

Normalized data sets were entered into a random effects general linear model analysis for the pre-meal and post-meal scans to compare high-calorie vs. non-food contrasts. Resulting statistical maps were corrected for multiple comparisons using whole brain cluster-based threshold correction^45,46^. This cluster-correction approach allows for correction of multiple comparisons to reduce Type I errors, while enabling the detection of true activations by exploiting the theory that areas of activation tend to stimulate changes over spatially contiguous groups of voxels vs. over sparsely isolated voxels. More specifically, the cluster correction was performed using the “ClusterThresh” Plugin with 1000 MonteCarlo simulations for each contrast map in BrainVoyager. We used an initial (uncorrected) threshold of *p* < 0.001 to *p* < 0.005 and a minimum contiguous cluster correction applied to each contrast map ranging from 10 to 68 voxels (260–798 mm^3^) to provide a family-wise error correction at *p* < 0.05.

To visualize effects, we conducted secondary analyses, whereby the magnitude of the BOLD effect (i.e., the mean *β*-value) was extracted for each participant for statistically significant regions (as identified in the primary analyses described above). Then, one-way analyses of variance were performed using the Statistical Package for Social Sciences v25.0 (Chicago, IL, USA) to examine the differences between high-calorie vs. non-food contrasts for each region. We also explored potential correlations between mean changes in brain activation (i.e., mean *β*-values) in regions from the high-calorie vs. non-food contrast that were significantly different between and within obesity groups (Class III vs. Class I/II) and food preference (“liking”) scores, as well as relevant meal consumption variables.

## Results

Characteristics of the study population are shown in Table 1. The majority of the obese endometrial cancer survivors were Caucasian and, on average, were ~61 years old. Approximately 45% had Class I or Class II obesity and 55% had Class III obesity at baseline with a mean BMI of 41.8 ± 8.2 kg/m^2^. As expected, hunger rankings post-meal were lower than pre-meal but were not significantly different between Class I/II and Class III obesity groups. Food preference (“liking”) ratings for high-calorie foods were higher in Class III (4.1 ± 0.5) compared with Class I/II obese patients (3.9 ± 0.5; *p* = 0.04). Class III obese patients consumed more total calories (584.0 ± 173.9 vs. 506.5 ± 142.5; *p* = 0.03) and more total fat (27.5 ± 10.0 vs. 23.5 ± 8.2; *p* < 0.05) and protein (38.6 ± 13.4 vs. 31.6 ± 9.5; *p* < 0.01) in the luncheon meal compared with Class I/II obese patients.

**Table 1:** Characteristics of endometrial cancer survivors with obesity seeking weight loss.

Characteristic	Total population (*n* = 85)	Class I and II obese (*n* = 38)	Class III oobese (*n* = 47)
Age (years)	59.7 (9.1)	61.7 (9.2)	58.1 (8.8)
Caucasian	78 (91.8%)	35 (91.5%)	43 (92.1%)
BMI (kg/m^2^)	41.8 (8.2)	34.6 (2.8)	47.6 (6.4)^a^
Pre-meal hunger	4.7 (2.3)	4.8 (2.4)	4.7 (2.4)
Post-meal hunger (“satiety”)	0.5 (0.8)	0.6 (0.9)	0.4 (0.7)
Meal: total energy (Kcal) Consumed	549.3 (164.4)	506.5 (142.5)	584.0 (173.9)^a^
Meal: % Kcal from fat consumed	40.9 (6.2)	40.6 (6.2)	41.1 (5.9)
Meal: total fat (g) consumed	25.7 (9.4)	23.5 (8.2)	27.5 (10.0)^a^
Meal: % Kcal from carbohydrate consumed	33.2 (7.9)	33.4 (8.2)	31.5 (7.7)
Meal: total carbs (g) consumed	45.1 (15.0)	43.3 (14.8)	46.6 (15.1)
Meal: % Kcal from protein consumed	26.8 (5.1)	26.0 (4.8)	27.4 (5.2)
Meal: total protein (g) consumed	35.5 (12.3)	31.6 (9.5)	38.6 (13.4)^a^
High-calorie food preference (“liking”)	3.98 (0.47)	3.86 (0.46)	4.08 (0.46)^a^
Low-calorie food preference (“liking”)	3.97 (0.46)	4.06 (0.41)	4.07 (0.50)

^a^*p*-value < 0.05 (Class III vs. Class I/II).

**Fig. 1:**
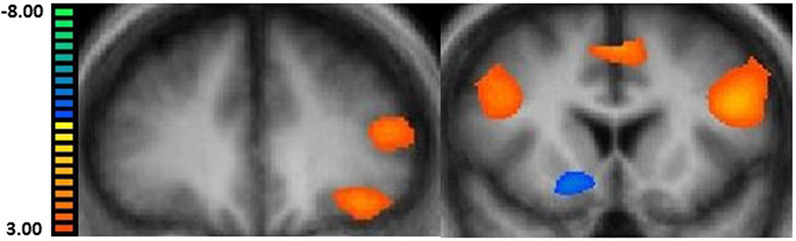
Neural activations from the high-calorie food vs. non-food visual cue contrast at baseline in obese endometrial cancer survivors seeking weight loss. Left panel (fasted pre-meal condition): increased activation in dorsolateral prefrontal cortex (DLPFC) and orbitofrontal cortex (OFC) (coronal view). Right panel (post-luncheon meal condition): increased activation in DLPFC (bilateral) and medial frontal gyrus (MFG) cortex and decreased activation in putamen (coronal view).

Among all endometrial cancer survivors with obesity, we observed several regions with differential neural activations for the high-calorie vs. non-food contrast (Table 2). In the pre-meal condition (fasted state), we found significant increased activation when comparing high-calorie vs. non-food cues in regions including the OFC (Brodmann Area (BA) = 47; *x* = −35, *y* = 32, *z* = −6) and DLPFC (BA = 46; −42, 32, 17) (Fig. 1, left panel). When comparing high-calorie vs. non-food images in the post-meal (fed) state, we found significant increased activation in several regions including the precuneus (Bilateral; BA = 39; 30, −66, 32), DLPFC (BA = 46; −42, 17, 25), and MFG (BA = 6; −4, 13, 46) (Fig. 1, right panel). Although not anticipated, we observed significant decreased activation in the post-meal condition for the high-calorie vs. non-food contrast in the posterior cingulate (Bilateral; BA = 30; 18, −60, 12), fusiform gyrus (BA = 37; −27, −42, −11), and putamen (15, 9, −6) (Fig. 1, right panel).

**Table 2:** Neural activations for high-calorie (HC) vs. non-food object (NF) visual food cues at baseline in endometrial cancer survivors with obesity seeking weight loss (*n* = 85).

	Pre-meal (fasted)					Post-meal (fed/satiated)				
	Peak voxel					Peak voxel				
Brain region (hemisphere)	*x*	*y*	*z*	Cluster size*	*t*	*x*	*y*	*z*	Cluster size*	*t*
**↑**Activation (HC > NF)										
OFC (BA = 47)	−35	32	−6	566	3.21					
DLPFC (BA = 46)	−42	32	17	667	3.13					
Precuneus (bilateral; BA = 19)						33	−64	37	684	3.37
Precuneus (bilateral; BA = 39)						30	−66	32	568	4.06
DLPFC (bilateral; BA = 46)						42	17	25	578	4.28
DLPFC (bilateral; BA = 9)						−41	13	26	433	5.39
Medial frontal gyrus (BA = 6)						−4	13	46	509	3.56
**↓**Activation (HC < NF)										
Posterior cingulate (BA = 30)	15	−54	13	647	3.90					
Parahippocampal gyrus (BA = 36)	27	−42	−9	566	3.50					
Insula (BA = 13)	36	−21	23	609	3.41					
Precuneus (BA = 7)	−6	−67	43	130	3.48					
Fusiform gyrus (BA = 37)	−27	−44	−11	588	4.48					
Precuneus (BA = 7)						15	−46	52	611	3.66
Posterior cingulate (bilateral; BA = 30)						18	−60	12	778	4.59
Putamen						15	9	−6	572	3.26
Parahippocampal gyrus (BA = 36)						27	−42	−7	639	3.86
Fusiform gyrus (BA = 37)						−27	−42	−11	714	4.67

*x*, *y*, *z* = coordinates in Talairach space; *t*-statistic for peak voxel.

^a^Cluster size is reported in mm^3^. Results are whole brain cluster corrected for multiple comparisons; threshold *α* < 0.05.

**Fig. 2:**
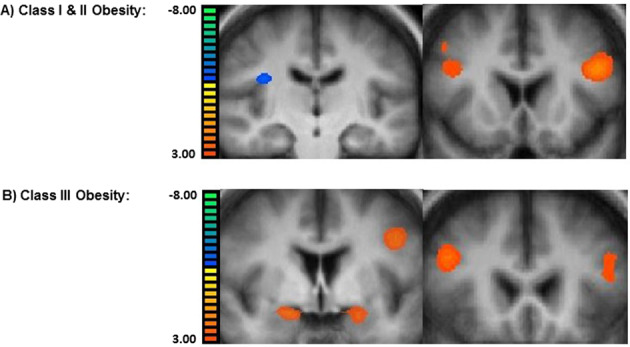
Neural activations from the high-calorie food vs. non-food visual cue contrast at baseline in endometrial cancer survivors seeking weight loss by obesity class. **a** Class I and II Obese: left panel (pre-meal): decreased activation in the insula (coronal view); right panel (post-meal): increased activation in dorsolateral prefrontal cortex (DLPFC; bilateral) (coronal view). **b** Class III Obese: left panel (pre-meal): increased activation in parahippocampal gyrus (bilateral) and precentral gyrus (coronal view); right panel (post-meal): increased activation in DLPFC and precuneus (coronal view).

When we stratified by obesity class, we observed differences in the neural activation patterns by class (Table 3). Within the Class I/II obese group, we did not observe significant increased activation in any region after cluster correction in the fasted state for the high-calorie vs. non-food contrast; however, we observed decreased activation in several regions including the insula (BA = 13; 33, −16, 22; Fig. 2a, left), parahippocampal gyrus (BA = 36; 27, −40, −10), and posterior cingulate (BA = 30; −42, 32, 17). Within the Class I/II obese group in the fed state, we observed increased activation in the DLPFC (BA = 46; −42, −34, 13; Fig. 2a, right) and decreased activation in the parahippocampal gyrus (bilateral; BA = 36; −27, −42, −10). Within the Class III obese group in the fasted state, we found significant increased activation when comparing high-calorie vs. non-food images in several regions including the OFC (BA = 47; −39, 35, −5), DLPFC (BA = 46; −42, 32, 17), precuneus (BA = 31; −27, −72, 28), parahippocampal gyrus (bilateral; BA = 34; 18, 0, −14), and precentral gyrus (BA = 6; *x* = −42, *y* = 2, *z* = 32) (Fig. 2b, left). In the fed state in the Class III group, we observed increased activation in the DLPFC (bilateral; BA = 46; 42, 21, 26; Fig. 2b, right) and precuneus (bilateral; BA = 7; −27, −64, 33), and decreased activation in the caudate (9, 8, −5) and posterior cingulate (BA = 30; 19, −59, 13).

**Table 3:** Neural activations in response to high-calorie food (HC) vs. non-food object (NF) visual cues at baseline in endometrial cancer survivors with obesity seeking weight loss by obesity class.

		Pre-meal (fasted)					Post-meal (fed/satiated)				
		Peak voxel					Peak voxel				
	Brain region (hemisphere)	*x*	*y*	*z*	Cluster size*	*t*	*x*	*y*	*z*	Cluster size*	*t*
**↑**Activation	Class III Obesity										
(HC > NF)	Parahippocampal gyrus (bilateral; BA = 34)	18	0	−14	521	3.06					
	Anterior PFC (BA = 10)	−27	57	18	453	3.01					
	OFC (BA = 47)	−39	35	−5	453	4.25					
	Precuneus (BA = 31)	−27	−72	28	635	3.58					
	Fusiform gyrus (BA = 37)	−42	−58	−10	493	3.82					
	DLPFC (BA = 46)	−42	32	17	626	3.89					
	Precentral gyrus (BA = 6)	−42	2	32	722	3.84					
	Precuneus (bilateral; BA = 19)						33	−64	40	615	3.53
	Precuneus (bilateral; BA = 7)						−27	−64	33	655	3.76
	DLPFC (bilateral; BA = 46)						42	21	26	605	3.38
	DLPFC (BA = 9)						−45	7	29	660	4.05
	Class I and II Obesity DLPFC (Bilateral; BA = 46)						−42	−34	13	495	4.07
↓Activation	Class III Obesity										
(HC < NF)	Caudate						9	8	−5	456	3.23
	Posterior cingulate (BA = 30)						18	−59	13	443	3.66
	Superior temporal gyrus (BA = 42)						55	−28	13	401	3.19
	Culmen						−24	−43	−11	563	3.54
	Superior temporal gyrus (BA = 22)						−66	−19	4	336	3.36
	Class I and II Obesity										
	Lingual gyrus (BA = 17)	−12	−90	−1	232	4.98					
	Parahippocampal gyrus (bilateral; BA = 36)	27	−40	−10	231	6.50	27	−42	−10	600	3.39
	Insula (BA = 13)	33	−16	22	244	4.90					
	Inferior parietal lobule (BA = 40)	33	−38	47	269	4.46					
	Precuneus (BA = 7)	−9	−69	42	252	4.51					
	Posterior cingulate (BA = 30)	−18	−61	12	258	4.48					

Results are whole brain cluster corrected for multiple comparisons; threshold *α* < 0.05.

World Health Organization (WHO) Obesity Classifications: Class I and II: BMI ≥ 30.0 to <40.0 kg/m ^2^; Class III: BMI ≥ 40.0 kg/m^2^.

When comparing activations between obesity groups (Class III vs. Class I/II) in the regions discussed above, only two regions were statistically significantly different between groups in the pre-meal condition. We found significantly higher activation in the DLPFC in Class III compared to Class I/II (*β* = 0.11 ± 0.03 vs. 0.02 ± 0.04; *p* = 0.04) and significantly lower activation in the insula in Class III compared with Class I/II (*β* = −0.01 ± 0.02 vs. −0.09 ± 0.03; *p* = 0.03). Higher activation in the OFC was marginally significant in Class III compared with Class I/II (*β* = 0.13 ± 0.04 vs. 0.03 ± 0.05; *p* = 0.09) in the pre-meal condition. We observed no statisticaaly significant differences in the post-meal condition between Class III and Class I/II patients.

We also explored potential correlations between activation regions that were significantly different between and within obesity classes in the high-calorie vs. non-food contrast in the fasted state and food preference (“liking”) scores, as well as meal consumption variables. We found no significant correlations between brain activation regions that were statistically significantly different between obesity class groups (Class III vs. Class I/II: DLPFC; insula) and high-calorie liking scores or between these regions (DLPFC; insula) and total calories, protein and fat consumed in the meal. However, we observed statistically significant correlations in brain regions that were differentially activated within obesity class in the high-calorie vs. non-food contrast, and meal consumption and food liking scores. In patients with Class III obesity post-meal, we found that higher activation in the DLPFC was positively correlated with the amount of carbohydrate consumed in the meal (*r* = 0.32; *p* = 0.03) and inversely correlated with fat consumed (*r* = −0.29; *p* = 0.04). In addition, post-meal in Class III patients, higher activation in the DLPFC was inversely correlated with fruit and vegetable-liking scores (*r* = −0.29; *p* < 0.05). In Class I/II patients post-meal, we found higher activation in the DLPFC was positively correlated with the amount of carbohydrate consumed (*r* = 0.33; *p* < 0.05) and marginally inversely correlated with fruit and vegetable-liking scores (*r* = −0.28; *p* = 0.09).

## Discussion

We found increased activation in several regions previously associated with food-related reward circuitry when comparing high-calorie to non-food object images in the fasted state including in the DLPFC in Class III and Class I/II obese patients and, that the activation in the DLPFC was significantly higher in Class III compared to Class I/II patients. In addition, we found, unexpectedly, a decreased activation in the insula in the fasted state, which was significantly lower in Class I/II compared to Class III obese patients. Furthermore, we found that the increased activation in the DLPFC persisted in both Class III and Class I/II obese patients but the difference in activation between the two groups post-meal was not statistically significantly different. In patients with Class III and Class I/II obesity, we found that higher activation in the DLPFC (post-meal) was positively correlated with the amount of carbohydrate consumed in the luncheon meal. In addition, in patients with Class III obesity, we observed that higher activation in the DLPFC post-meal was inversely correlated with the amount of fat consumed and fruit and vegetable-liking scores.

These findings are generally consistent with prior studies in adults with obesity without cancer and our prior pilot study in endometrial cancer survivors with obesity. In our prior pilot study, we found increased activation in the fasted state when comparing high-calorie vs. non-food cues in the DLPFC and OFC, which is consistent with our findings here in a larger population of endometrial cancer survivors^40^. However, we also observed increased activation in the MFG in the fasted state in our pilot study^40^, which we did not observe here in the larger population of endometrial cancer survivors with obesity. In the fed state in the pilot study, we found significant increased activation in the thalamus, posterior cingulate, and precuneus^40^, and, here, in our larger population, observed increased activation in the precuneus, as well as the DLPFC and MFG. Prior studies in adults with obesity without cancer have shown increased activation in the fasted and fed states in the DLPFC, OFC, precuneus and MFG^47,48^. Moreover, multiple studies have now reported on the significant overlap in the response to visual food and drug cues in several brain regions including the DLPFC, precuneus, and MFG^49,50^.

In patients with Class III and Class I/II obesity, we found that higher activation in the DLPFC (post-meal) was positively correlated with the amount of carbohydrate consumed in the luncheon meal. In addition, in patients with Class III obesity, we observed that higher activation in the DLPFC post-meal was inversely correlated with the amount of fat consumed and fruit and vegetable-liking scores. These findings suggest that increased activation in response to high-calorie visual food cues in attention and inhibitory control regions (DLPFC) persists even after eating to satiation in patients irrespective of obesity class and, that increased attention and inhibitory control may be associated with an increased intake of carbohydrates and a lower preference for fruits and vegetables. Only a few studies evaluating the neural response to visual food cues in adults with obesity have reported on correlations between regions with significant differential activation and total energy or macronutrient intake. In one study, differential activation in the dorsal striatum in response to food vs. non-food cues was positively correlated with the percentage of calories consumed from added sugar (as measured by dietary recalls during the prior 24 h); however, there was no discussion regarding correlations between frontal region activations and added sugar consumption^51^. Sucrose consumption compared with water and non-sugar-sweetened (caloric) beverages was found to illicit higher activation in the DLPFC^52^ but these authors did not report correlations between the DLPFC activation and the amount sucrose ingested. Additional studies examining correlations between brain regions with differential activation to visual food cues and meal consumption variables in adults within and between different classes of obesity are needed.

When we stratified by obesity class, we found increased activation in the fasted state in several regions including the DLPFC in Class III and Class I/II obese groups, and that the activation in the DLPFC was significantly higher in Class III compared with Class I/II. Furthermore, we found increased activation in the DLPFC that persisted in Class III and Class I/II after eating but the difference in activation between the two groups was not significantly different in the fed state. In the fasted and fed state, we found increased activation in precuneus in Class III but not in Class I/II. We observed increased activation in the precentral gyrus in Class III but only in the fasted state. Unexpectedly, we observed decreased activation in the insula in the fasted state, which was significantly lower in Class I/II compared to Class III. Furthermore, we observed a decreased activation in the PC in the fasted state in Class I/II and, in the fed state in Class III patients; however, the activation in the PC was not significantly different between groups.

As this is the first study to formally evaluate food cue response between different classes of obesity, we are not able to directly compare our findings to prior literature. However, increased activation in the DLPFC has been found in numerous studies in obese adults in fasted and fed states and has been associated with eating restraint^47,48^. Projections from the amygdala may modulate activity in the posterior visual areas such as the precuneus to increase the salience of cues through attentional bias toward the food cues^49,53^; thus, the precuneus may be an important region for relaying information in response to visual cues. Increased activation in the precentral gyrus may be related to the preparatory steps for motor planning and anticipatory movement for the ultimate ingestion of food^33^. Food selection is guided by the visual system and the sight of food invokes physiological, emotional, and cognitive responses which may enhance the desire to want to ingest certain foods^48,54–56^. Thus, the differential activation observed collectively in these regions could suggest potentially altered decision-making of food intake based on emotional and integrative control and motor systems (precuneus and precentral gyrus)^57^. The insula is a region associated with taste information processing and the PC associated with memory^28,30^; thus, the decreased activation we observed in the fasted state among Class I/II obese patients in these regions was not expected or consistent with prior reports and requires further investigation. The majority of prior studies examining response to visual food cues have been conducted in adults without cancer who are substantially younger (most subjects were under the age of 40), which could contribute to some inconsistencies^58^. In addition, the reproducibility of neuroimaging studies may be affected by differences not only in the characteristics of the study population but by differences in study designs, tasks, stimuli, hunger states (fasted/fed), and sample size. Nevertheless, meta-analyses of neuroimaging studies involving visual food cues have shown that there is a “moderate” level of concordance between studies^48^. As this is only the second study in cancer survivors with obesity, it is difficult to address consistency, particularly since our first study involved such a small number of patients^40^. Additional studies evaluating visual food cues in cancer survivors with obesity are needed, especially given that cancer survivors with obesity are at a higher risk of death^10,15,16^ and undergo treatments that could further disrupt their homeostatic and non-homeostatic energy systems making weight loss even more challenging^59,60^.

Based on the collective evidence, we purport that future behavioral weight loss interventions, particularly in adults with severe obesity, should aim to enhance inhibitory control in frontal brain regions. Interventions could potentially integrate “attention training”^61^ and/or transcranial direct current stimulation targeted to the DLPFC^62,63^ in conjunction with other weight loss strategies. Our findings from this study together with prior reports suggest that targeting the DLPFC may be potentially beneficial in all obese adults but may be particularly important in treating Class III or severely obese patients.

Advantages of our study include a relatively large sample size that enabled stratification by obesity class/severity. Disadvantages include that we did not have a population of non-cancer adults with Class I/II and Class III obesity with which to directly compare to. In addition, no prior studies have formally evaluated neural response to food cues in Class I/II compared to Class III patients; thus, it is difficult to assess consistency. Other disadvantages include the general issues with reproducibility in neuroimaging studies mentioned above, which are affected by differences in study design, specific stimuli and hunger states that could contribute to differences. Nevertheless, the increased activation we observed in the DLPFC, a brain region associated with cognitive control/inhibition, is consistent with the summative literature in adults with obesity; however, the decreased activation in the insula, a region associated with taste information processing, which was stronger in Class I/II patients was unexpected and requires additional investigation.

In summary, our results provide novel insights on food cue response between different classes of obesity and highlight the importance of targeting the DLPFC in weight loss interventions, particularly in severely obese patients. Additional studies examining food-related neural circuitry between different classes of obesity are needed.
